# High-Frequency Repetitive Transcranial Magnetic Stimulation Applied to the Parietal Cortex for Low-Functioning Children With Autism Spectrum Disorder: A Case Series

**DOI:** 10.3389/fpsyt.2019.00293

**Published:** 2019-05-09

**Authors:** Yingxue Yang, Hongxing Wang, Qing Xue, Zhaoyang Huang, Yuping Wang

**Affiliations:** ^1^Department of Neurology, Xuanwu Hospital, Capital Medical University, Beijing, China; ^2^Beijing Key Laboratory of Neuromodulation, Beijing, China; ^3^Center of Epilepsy, Beijing Institute for Brain Disorders, Laboratory of Brain Disorders, Capital Medical University, Ministry of Science and Technology, Beijing, China

**Keywords:** repetitive transcranial magnetic stimulation, autism spectrum disorder, inferior parietal lobule, social relating, language

## Abstract

**Background:** Repetitive transcranial magnetic stimulation (rTMS) is a safe and efficacious technique to stimulate specific areas of cortical dysfunction in several neuropsychiatric diseases; however, it is not known whether high-frequency rTMS (HF-rTMS) over the left inferior parietal lobule, in low functioning children with autism spectrum disorder (ASD), improves core symptoms.

**Method:** Eleven low-functioning children with ASD completed two separate HF-rTMS treatment courses, 6 weeks apart. Each treatment course involved five 5-s trains at 20 Hz, with 10-min inter-train intervals, on left inferior parietal lobule each consecutive weekday for a 3-week period (15 treatments per course). Subjects were assessed at five time points: immediately before and after the first HF-rTMS course, immediately before and after the second HF-rTMS course, and 6 weeks after the second rTMS treatment course. Treatment effectiveness was evaluated using the Verbal Behavior Assessment Scale (VerBAS) and Autism Treatment Evaluation Checklist (ATEC). The latter test consists of four subtest scales: Language, Sociability, Sensory, and Behavior. In addition, daily treatment logbooks completed by parents were considered as one of the outcome measures.

**Results:** Participants showed a significant reduction in language- and social-related symptoms measured by ATEC from pretreatment to the 6-week follow-up after the second treatment course. Moreover, some possible improvements in imitation and cognition were reported by caregivers.

**Conclusions:** Our findings suggest that HF-rTMS over the left parietal cortex might improve core deficits in low-functioning children with ASD.

## Introduction

Autism spectrum disorder (ASD) is characterized by deficits in social communication and stereotyped behaviors (1). Despite the spectrum’s extreme heterogeneity, deficits in social cognition, including reduced social responsiveness, difficulty interacting with others, and recognizing others’ intentions and emotions, are core features of ASD (1).

Dysfunction of the mirror neuron system (MNS) has been postulated in the pathophysiology of ASD (2). Mirror neurons are visuomotor cells that discharge not only when an individual performs a particular action but also when a similar action is observed (2, 3). The mirror neuron system (MNS) enables individuals to interpret motor acts of others and promotes the development of social cognition, such as emotion and empathy (3). Besides, MNS facilitates motor coordination and participates in memory, speech, and action planning (3–5).

MNS predominantly comprises the inferior frontal gyrus, inferior parietal lobule (IPL), and posterior superior temporal sulcus (6). Recent studies suggest that a dysfunction of the MNS might generate social and cognitive impairments related to ASD (7). It has been found that motor neurons of the IPL can code motor goals (8) and process the congruence between the executed and the observed motor act (8, 9). It has also been demonstrated that any damage to the parietal cortex affects the imitation or understanding of an observed action (10). Therefore, IPL is a likely neurobiological target for the treatment of ASD.

Repetitive transcranial magnetic stimulation (rTMS) offers a noninvasive approach for modifying cortical excitability. It potentially evokes a short-term functional reorganization in the brain (11). Effects of rTMS are not limited to the primarily stimulated cortex, because of anatomical and functional connections of cortical regions within a distributed network (11–13). Studies have suggested that low-frequency rTMS (<1 Hz) decreases cortical excitability, whereas HF-rTMS (>5 Hz) increases it (14, 15). Neuroenhancement of MNS in typically developing individuals has been reported using high-frequency (20 Hz) rTMS (HF-rTMS) (16).

A limited number of research studies have evaluated the therapeutic effects of rTMS in ASD. For example, it has been reported that applying low-frequency rTMS to the dorsolateral prefrontal cortex causes a reduction in stereotypical behaviors (17). Stimulation of IPL, however, has not been undertaken in ASD. Moreover, few studies investigated the effects of rTMS in children with ASD and intellectual disability. In the present study, we examined the effects of HF-rTMS on IPL in autism associated with severe intellectual disability. We hypothesized that HF-rTMS application to IPL would result in improvements in social functioning.

## Methods

### Participants

Thirteen participants with ASD (age range 3–12 years) were recruited from Xuanwu Hospital, Capital Medical University, Beijing, China. Diagnosis was made by an experienced physician according to the *Diagnostic and Statistical Manual of Mental Disorders, Fifth Edition* (DSM-V) (1), and further confirmed with the Autism Diagnostic Interview–Revised (ADI-R) (18) and Autism Behavior Checklist (ABC) (19), administered by physicians trained to clinical reliability. Cases with a personal and family history of seizure, the presence of metal implants, were excluded. No subjects were on psychotropic medications. All 13 participants had IQ <70 measured by the Wechsler Intelligence Scale for Children, Fourth Edition (WISC-IV) (20). Two patients withdrew from the study during the first course of the treatment due to family reasons. The data of these two individuals were excluded in the final sample. Participant information is summarized in **Table 1**.

**Table 1:** Participant demographics.

Participant number	Gender	Age (years)	ABC (scores)
1	Male	7	73
2	Male	7	93
3	Male	6	63
4	Female	5	70
5	Female	5	71
6	Male	11	64
7	Female	9	82
8	Female	9	86
9	Male	4	81
10	Male	3	85
11	Male	12	107

ABC, Autism Behavior Checklist.

This study had the approval of the ethics committee of Xuanwu Hospital, and all participants’ parents provided written informed consent before the study.

### Procedures

A Magstim Super Rapid stimulator (The Magstim Company Ltd., Whitland, UK) connected to a 70-mm figure-of-eight coil was used to perform rTMS. The stimulation was applied on the left IPL [electrode P3 on the electroencephalography (EEG) cap] (21). Participants completed two separate courses that were 6 weeks apart. Each treatment course consisted of five 5-s trains at 20 Hz, with 10-min intertrain intervals, each consecutive weekday for a 3-week period (15 treatments per course). Because most participants could not participate in motor threshold assessments, we referred to the resting motor thresholds (RMT) measured in children (7–13 years old) with Tourette syndrome and children (8–13 years old) with attention deficit hyperactivity disorder in our laboratory. RMT of these children mostly ranged from 40% to 50%. Thus, the stimulation intensity was set uniformly at 50% of stimulator output.

Subjects were evaluated at five time points: immediately before the first HF-rTMS course (“pre-1”), immediately after the first HF-rTMS course (“post-1”), immediately before the second HF-rTMS course (“pre-2”), immediately after the second HF-rTMS course (“post-2”), and 6 weeks after the second rTMS treatment course (6 weeks later, “6wl”). Treatment effectiveness was assessed using the Verbal Behavior Assessment Scale (VerBAS) (22) and Autism Treatment Evaluation Checklist (ATEC) (23). ATEC consists of four subtest scales: Scale I (Speech/Language/Communication), Scale II (Sociability), Scale III (Sensory/Cognitive awareness), and Scale IV (Health/Physical/Behavior) (23). In addition, daily logbooks completed by caregivers were considered as one of the outcome measures.

**Figure 1:**
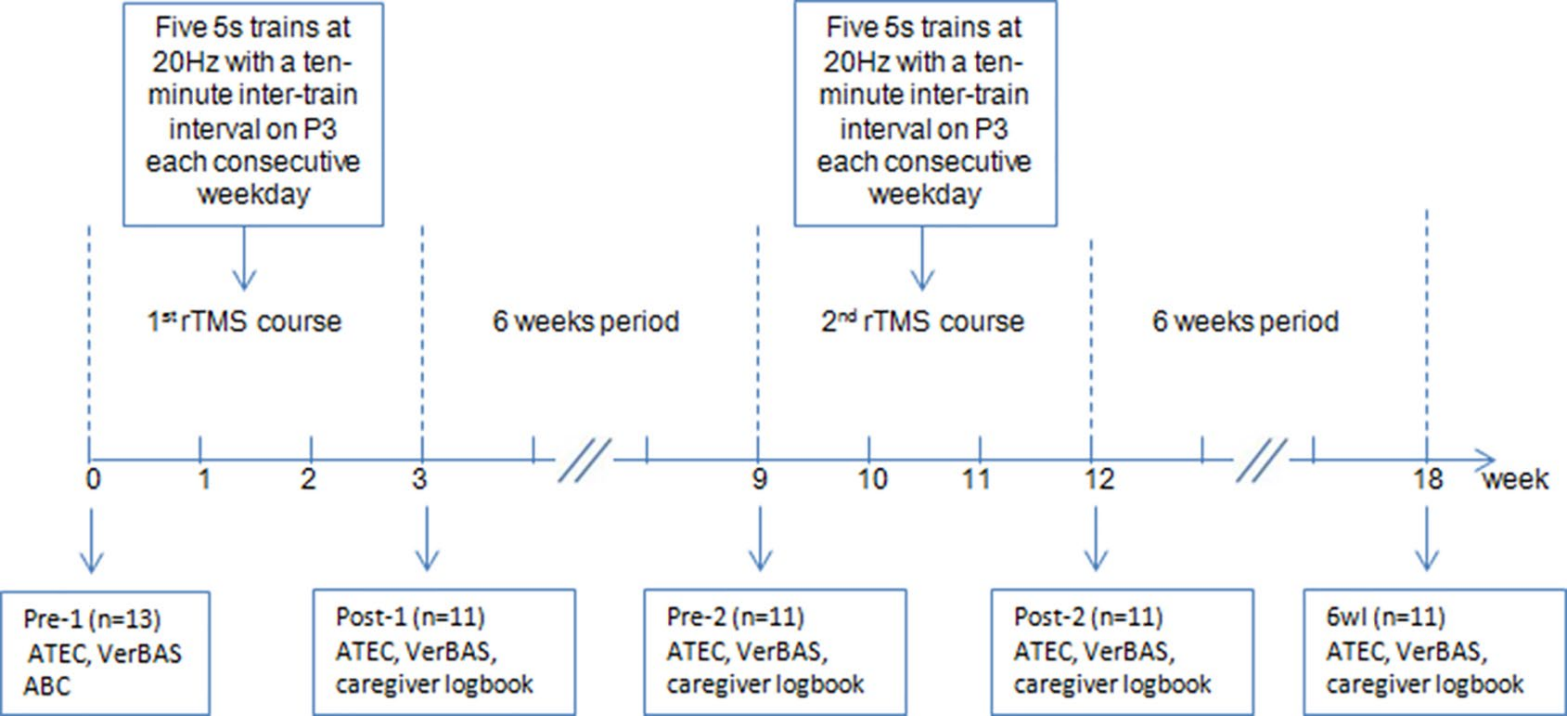
Flow diagram of the experiment. Note: ATEC, Autism Treatment Evaluation Checklist; VerBAS, Verbal Behavior Assessment Scale; ABC, Autism Behavior Checklist; P3, left parietal electrode.

Moreover, we monitored any side effects during the stimulation courses and instructed caregivers to report any side effects they noted during and after treatment. The protocol flow diagram is shown in **Figure 1**.

### Data Analysis

Statistical analysis was completed using Statistical Product and Service Solutions (SPSS) software (version 19.0, SPSS Inc., IL, USA). A *P* value <0.05 was considered significant for all analyses. One-way ANOVA with repeated measures was used to examine differences in the effects of HF-rTMS on the four ATEC scale scores, as well as VerBAS scores among different time points (pre-1 vs. post-1 vs. pre-2 vs. post-2 vs. 6wl). Bonferroni correction was used to adjust *P* values in *post hoc* analyses.

## Results

Eleven individuals (7 boys, 4 girls) with a mean age of 7.09 ± 2.88 years completed the two treatment courses and follow-up assessments. There were no reports of serious adverse events. Transient irritability during or after HF-rTMS was reported in three cases by caregivers (**Table 2**). One participant became irritable during the first 3 days in each treatment course. Another was more emotional after the second treatment course and recovered in 5 days. A third was hyperactive and irritable during the first 5 days of the first course.

**Table 2:** Side effects reported by caregivers during and after high-frequency repetitive transcranial magnetic stimulation (HF-rTMS) treatment courses.

Participant no.	Side effects
1	Irritable during the first 3 days of each treatment course. For example, crying for a longer period of time if a need was not immediately met.
2	More emotional and restless after the second course, which recovered in 5 days; occasionally hitting the head against the wall in episodes of anger.
9	Hyperactive, irritable during the first 5 days of the first course.

The repeated-measures ANOVA revealed significant changes over time in the ATEC language scale [*F*(4,50) = 2.685, *P* = 0.042] and ATEC social scale [*F*(4,50) = 2.636, *P* = 0.045]. The least significant difference (LSD) method was used in *post hoc* analysis. The ATEC language scale significantly decreased from “pre-1” to “post-2” (*P* = 0.048) and from “pre-1” to “6wl” (*P* = 0.003). There was also a significant reduction in ATEC social scale from “pre-1” to “post-2” (*P* = 0.021) and from “pre-1” to “6wl” (*P* = 0.005).

However, after *P* value correction by Bonferroni method, the difference between “pre-1” and “post-2” did not achieve statistical significance in the ATEC language and social scales. The ATEC language scale significantly decreased from “pre-1” to “6wl” (*P* = 0.025) (lower ATEC scores reflect reduced impairments). The ATEC social scale significantly decreased from “pre-1” to “6wl” (*P* = 0.048).

No statistically significant changes over time were found in ATEC sensory and cognitive awareness scale [*F*(4,50) = 0.234, *P* = 0.918], ATEC health and behavioral scale [*F*(4,50) = 0.398, *P* = 0.809], or VerBAS [*F*(4,50) = 1.086, *P* = 0.374]. Summary data for clinical measures were presented in **Table 3**.

**Table 3:** Autism Treatment Evaluation Checklist (ATEC) scale scores and Verbal Behavior Assessment Scale (VerBAS) scores at each assessment time point (mean ± SD).

	Pre-1	Post-1	Pre-2	Post-2	6wl
ATEC language scale	16.1 ± 5.3	13.6 ± 5.0	12.9 ± 4.2	12.1 ± 4.4	9.8 ± 4.1*
ATEC social scale	19.8 ± 7.5	17.0 ± 7.0	15.6 ± 5.6	13.6 ± 5.1	12.2 ± 4.7*
ATEC sensory and cognitive awareness scale	21.3 ± 5.0	20.1 ± 6.0	19.8 ± 6.0	19.4 ± 5.4	19.2 ± 5.8
ATEC health and behavioral problems scale	21.2 ± 8.2	19.5 ± 7.8	17.4 ± 7.3	18.2 ± 7.1	18.6 ± 7.7
VerBAS scale	30.6 ± 8.8	34.4 ± 10.2	34.9 ± 9.7	36.9 ± 9.4	38.6 ± 9.7

*Significantly different from “pre-1” (P < 0.05).

For ATEC, lower scores reflect reduced impairments.

For VerBAS, higher scores reflect reduced impairments.

6wl, 6 weeks later.

According to the clinical observations and caregiver reports, HF-rTMS might be more effective in male children than in female children. Most caregivers reported their children displayed possible improvements in imitation and cognition (e.g., language imitation and behavior imitation) after the HF-rTMS treatments. We summarized some improvements from caregiver logbooks in 11 participants in **Table 4**.

**Table 4:** Improvements in the quality of life of 11 participants, following HF-rTMS treatments. Records from caregiver’s logbooks.

No.	Posttreatment assessment			
	Language	Social skills	Imitation, cognition, learning, fine motor skills	Behaviors and emotions
1	Increased active language, e.g., initiatively saying “go home” after treatment	More eye contact. Showed greater affection toward family members. Helped parents do housework. Willing to play games with other children	Enhanced learning and imitation ability. Accepted new knowledge faster than before. Improved comprehension and execution	A slight decrease in repetitive behavior
2	Decreased self-talk	Willing to play games with other children. Taking the bus quietly instead of shouting, especially when there were no seats available. Showed greater affection toward family members. Aware of location of parents when taking the bus	Improved attention and comprehension. Could understand the explanation of game rules. Improved imitation. Showed more patience with writing and painting. Improved fine motor skills.	A slight decrease in repetitive behavior
3	Louder voice and clearer speech. Expanded vocabulary. Could say “no” to express unwillingness. Increased active language, e.g., naming objects he recognized on TV	N/A	Improved attention, comprehension, and imitation. Improved discernment of color and shape. Improved fine motor skills	More physically active
4	Speaking loudly and clearly	Closer to parents. More eye contact	Faster reaction time. Could understand some instructions	Laughed more than before
5	Louder speech. Increased active language, e.g., actively calling “Dad,” “Mom” (first time occurrence since birth). Often says “Ah,” with pitch variation	N/A	Faster reaction time	Improvement in bad temper. More smiles than before
6	N/A	Willing to play games with other children. More understanding of surrounding environment, e.g., looking around when crossing the road. Quietly sitting for 2–3 h during a conference and applauding with others	Improved concentration and comprehension. Could understand and carry out two simultaneous instructions	N/A
7	Increased active speech, e.g. actively calling “Dad,” “Mom”	Closer to parents and sister. Willing to play games with other children	Could understand and carry out some instructions	Obvious decrease in frequency of crying
8	N/A	Closer to parents and sister; willing to play games with other children	Could understand and carry out some instructions	N/A
9	During the third week of the first treatment course, passive language imitation gradually increased. At the beginning of the second course, spontaneously imitated what parents and teachers said. Could answer some simple questions, such as his name, age, and parents’ names	More eye contact; willing to be together with family members	Improved comprehension, memory, and imitation	A slight decrease in repetitive behavior (not obvious)
10	Increased active language. Clearer speech. Could say five-to-six-word vs. two-to-three-word sentences before treatment. Could answer some simple questions, e.g., age, name, and what he liked to eat	Paid attention to other children when playing. Likes to be close to family. If parents go out, he would catch up or become unhappy	Improved comprehension, memory, and execution. Became interested in reading.	N/A
11	Reduced repetitive language. More accurate oral expression. Initiatively expressed his opinions, e.g., “I want to sit down,” when tired	Could wait in line and quietly ride public transportation	Improved learning and imitation. Could sometimes understand parents’ words	Greatly reduced impulsive and violent behaviors

N/A, not applicable.

## Discussion

This study provides preliminary evidence for the effectiveness and safety of HF-rTMS over the left IPL as a treatment option to improve core symptoms in low-functioning autism. Specially, HF-rTMS significantly reduced social and speech deficits as measured by ATEC and parents’ report. At the same time, children’s imitation and cognition might be improved following treatment.

The specific mechanisms underlying these effects may reflect specific neuroplastic effects associated with high-frequency stimulation and will require further investigation. Physiological experiments in humans indicate that HF-rTMS evokes long-term potentiation (LTP) of synaptic transmission (24, 25). These changes are not only restricted to the site of stimulation but also observed in a widespread cortical and subcortical network (26, 27). The use of HF-rTMS (20 Hz) to adaptively modulate properties of the MNS in humans has been reported in typically developing individuals (16).

From a neurophysiological perspective, we hypothesize that these clinical effects resulted from stimulation of IPL and associated MNS, which have been linked to ASD (7). Stimulation of the IPL may induce long-lasting changes in the excitability of regions within the MNS network (28). Such alterations may improve one’s understanding of social environment and may reinforce the capacity for imitation. Thus, an enhanced interpretation of social context may lead to improvements in language and social skills, as shown in the current trial (28).

Growing evidence suggests that dopaminergic dysfunction is implicated in the pathogenesis of ASD (29, 30). Human studies show that HF-rTMS of the frontal cortex induces the release of dopamine in the cortical, limbic, and striatal brain regions (31). In this study, HF-rTMS on parietal cortex might alter dopamine activity in specific brain regions, which is related to social cognition in ASD (30).

To the best of our knowledge, this study is the first attempt at HF-rTMS over the IPL in intellectually disabled individuals with ASD. According to clinical observations and caregiver reports, HF-rTMS in this trial might be more effective in boys than in girls. The underlying reasons for the significant gender disparities in treatment outcome are not clear. However, female individuals with ASD seem to exhibit lower IQ (32), more severe phenotypes (33), overall autistic symptoms (34), and psychopathological problems (35). Moreover, it is important to note that the four girls in our study were two sets of twins; thus, genetic factors may play a critical role in their pathogenesis.

It may be confusing that the clinical effects measured by scales did not turn up at the time point of “immediately after the treatment course.” We thought there may be two reasons to account for this. Firstly, the number of participants in our study was relatively small, which may have a great impact on statistical analysis. Despite the limited validity of parental reports as outcome measures, the improvements in social cognition and speech were indeed observed during and after the treatment course. However, the improvements were not reflected by statistical analysis, as we expected. Secondly, repeated sessions of HF-rTMS could produce remodeling with an increase in active synapses (36), which may be responsible for cumulative rTMS effects. This may explain why the difference achieved statistical significance only at the time point of “six weeks after the second treatment course.”

Our study had several limitations that should be mentioned, including the fact that the study does not contain a control group (e.g., sham HF-rTMS), the conclusions are limited by the small sample size, limited validity of parental reports as outcome measures, and lack of neuroimaging and/or neurophysiological assessments. In future follow-up studies, large case–control clinical trials are necessary to explore the use of HF-rTMS as a unique treatment for improving core symptoms in ASD.

## Conclusion

Our original findings suggest that HF-rTMS on IPL has the potential to become a distinct therapeutic method aimed at treating core symptoms of ASD.

## Ethics Statement

This study was carried out in accordance with the ethics committee of Xuanwu Hospital with written informed consent from all subjects. All subjects gave written informed consent in accordance with the Declaration of Helsinki. The protocol was approved by the ethics committee of Xuanwu Hospital, Capital Medical University (LYS2017063).

## Author Contributions

YY participated in the design of the study, conducted the analyses, and wrote the manuscript; HW was involved in the study design, supervised the data analysis, and revised the manuscript; QX and ZH helped collected participants and coordinated the study; YW conceived and coordinated the design of the study and revised the manuscript. All authors read and approved the final manuscript.

## Funding

This research was supported by the National Natural Science Foundation of China (81801124) and the Beijing Municipal Hospital Research and Development Plan (PX2017069).

## Conflict of Interest Statement

The authors declare that the research was conducted in the absence of any commercial or financial relationships that could be construed as a potential conflict of interest.
